# Practice Facilitation to Support Primary Care Physicians With COVID-19 Vaccine Uptake: A Randomized Clinical Trial

**DOI:** 10.1001/jamanetworkopen.2025.9967

**Published:** 2025-05-14

**Authors:** Jennifer Shuldiner, Noor-Ul-Huda Shah, Stacey Bar-Ziv, David M. Kaplan, Michael E. Green, Ravninder Bahniwal, Isaac I. Bogoch, Dominik Alex Nowak, Laura Desveaux, Monica Taljaard, Justin Presseau, Holly O. Witteman, Aisha Lofters, Tara Kiran, Joe Mauti, Simran Gill, Archchun Ariyarajah, Jawad Chishtie, Denis Tsang, Mina Tradrous, Daniel Warshafsky, Jia Hu, Sabina Vohra-Miller, Noah Ivers

**Affiliations:** 1 Research and Innovation Institute, Women’s College Hospital, Toronto, Ontario, Canada; 2 Department of Family and Community Medicine, University of Toronto, Toronto, Ontario, Canada; 3 Institute for Health Policy Management and Evaluation, University of Toronto, Toronto, Ontario, Canada; 4 Ontario Health, Toronto, Ontario, Canada; 5 Departments of Family Medicine, Queen’s University, Kingston, Ontario, Canada; 6 Department of and Public Health Sciences, Queen’s University, Kingston, Ontario, Canada; 7 Division of Infectious Diseases, Toronto General Hospital, Toronto, Ontario, Canada; 8 Dalla Lana School of Public Health, University of Toronto, Toronto, Ontario, Canada; 9 Institute of Better Health, Trillium Health Partners, Mississauga, Ontario; 10 Clinical Epidemiology Program, Ottawa Hospital Research Institute and School of Epidemiology and Public Health, University of Ottawa, Ottawa, Ontario, Canada; 11 Department of Family and Emergency Medicine, Université Laval, Quebec City, Quebec, Canada; 12 Department of Family and Community Medicine and MAP Centre for Urban Health Solutions, St Michael’s Hospital, Unity Health Toronto, Toronto, Ontario, Canada; 13 Leslie Dan Faculty of Pharmacy, University of Toronto, Toronto, Ontario, Canada; 14 Office of the Chief Medical Officer of Health; 15 Department of Community Health Sciences, University of Calgary, Calgary, Canada; 16 ICES Central, Toronto, Ontario, Canada

## Abstract

**Importance:**

Recommendations by family physicians are associated with uptake of vaccines, but many family physicians had limited capacity to identify patients in their practice who might benefit from personalized vaccination counselling. Practice facilitation is an evidence-based method of supporting changes in primary care, but its role in supporting COVID-19 vaccination rates is unknown.

**Objective:**

To determine whether a multicomponent practice facilitation intervention would increase COVID-19 vaccine rates in the practices of family physicians with the largest number of unvaccinated patients.

**Design, Setting, and Participants:**

This 2-arm cluster-randomized clinical trial was conducted from November 15, 2021, to March 15, 2022, in Ontario, Canada’s most populous province. Data were obtained from the provincial vaccine registry and were linked to routinely used administrative databases. Six hundred family physicians whose practices had the largest number of unvaccinated rostered patients in the province were randomized 1:1 to a practice facilitation intervention or a control group. Eighteen were excluded because they were not practicing family physicians. Follow-up was completed March 31, 2022, and data were analyzed from March 2023 to May 2024. The primary analysis was by intention-to-treat; unit of analysis was the patient.

**Intervention:**

Practice facilitators offered physicians support to identify, reach out to, and counsel their unvaccinated patients.

**Main Outcomes and Measures:**

Any vaccine dose during a 4-month follow-up interval among rostered patients older than 12 years was used to calculate the rate of doses per 100 patients. A modified robust Poisson regression method was used to analyze intervention effects; intervention effect was estimated as relative risk (RR) using least square means differences with 95% CIs.

**Results:**

Of 582 physicians included in the analysis (median age, 58 [IQR, 52-66] years; 426 [73.2%] male), 292 were randomized to the control arm and 290 to the intervention arm. Only 84 physicians (29.0%) in the intervention arm accepted assistance from a practice facilitator. Mean numbers of doses of COVID-19 vaccines per 100 patients were 49.8 (95% CI, 48.8-50.9) in the intervention arm and 50.2 (95% CI, 49.2-51.2) in the control arm (adjusted RR, 0.99; 95% CI, 0.96-1.02).

**Conclusions and Relevance:**

In this study, practice facilitation to primary care clinics with high numbers of patients unvaccinated against COVID-19 was not associated with significant changes in vaccination uptake. Findings suggest the importance of ensuring that interventions target health services with high levels of both motivation and opportunity for improvement.

**Trial Registration:**

ClinicalTrials.gov Identifier: NCT05099497

## Key Points

**Question:**

Does a multicomponent practice facilitation intervention increase COVID-19 vaccination rates among practices of family physicians with the largest number of unvaccinated patients?

**Findings:**

In this randomized clinical trial of 582 physicians, the intervention had no significant impact on vaccination uptake. The adjusted relative risk for vaccine doses per 100 patients was 0.99, indicating no difference in vaccination rates between the intervention and control groups.

**Meaning:**

These findings suggest that future interventions should target health services with both high motivation and opportunity for improvement to be more effective.

## Teaser

This cluster-randomized clinical trial assesses whether availability of a multicomponent practice facilitation intervention for family physicians with large numbers of unvaccinated patients would increase COVID-19 vaccine uptake in Ontario, Canada.

## Introduction

One way to build vaccine confidence and increase uptake is through advice from a trusted health professional.^1,2^ Many Canadians consider family physicians their most trusted source of vaccine information.^1,2,3^ During the COVID-19 pandemic, there was an urgent need to vaccinate as much of the population as possible. To encourage vaccine uptake, a family physician would need the ability to identify patients who are unvaccinated and have the requisite knowledge, communication skills, and time to navigate questions about vaccinations.^4^ Unfortunately, the primary care environment has substantial time and staffing constraints.^5^ Furthermore, in Ontario, only 4% of the first vaccine does and 3% of the second vaccine dose were administered in primary care offices.^6^ Family physicians were therefore unable to foster opportunistic conversations in the same way they do for other vaccines.

In the summer of 2021, the Ontario government used the provincial registry of COVID-19 vaccinations to prepare reports for family physicians to provide data-driven insights to support their efforts in counselling patients around COVID-19 vaccine uptake. Two months after launching these reports, only 27% of family physicians had opened their report. Even in the best of times, asking family physicians to reach out to patients overdue for clinical care adds measurably to their workload, and this workload is amplified when recognizing that conversations with patients who are vaccine-hesitant can be emotionally fraught and challenging.^7^ Recognizing the value in providing external support to help family physicians work to their full scope,^8^ we developed an intervention to support family physicians in leveraging their vaccination reports for improved practice-level outcomes.

Practice facilitators^9^ use techniques to address gaps in care delivery,^10^ which may include connecting to outside resources, optimizing the use of electronic health records, implementing evidence-based practices, and addressing barriers to improve processes.^9^ These techniques can support family physicians to improve process flow, preventative and chronic care, and staff satisfaction within their clinics, ultimately resulting in better care for patients.^11,12,13,14,15,16^ To maximize population health impact in a system, it is desirable to provide facilitation to those practices that need it most, rather than to only those with time and capacity to volunteer for such initiatives. Therefore, we identified family physicians with the largest number of unvaccinated patients. We conducted a randomized clinical trial that tested whether offering practice facilitation for these family physicians would increase COVID-19 vaccine uptake among their patients.

## Methods

### Trial Design

We conducted a 2-arm pragmatic^17^ cluster-randomized clinical trial (Figure) to evaluate the effectiveness of the intervention in a clinical practice setting.^2^ A pragmatic trial was defined as a clinical trial testing effectiveness in routine care. Randomization occurred at the physician level, with patients grouped by their physician, rather than being individually randomized. The analysis was conducted at the patient level, but accounts for the clustering effect of physicians on patient outcomes.

**Figure.**
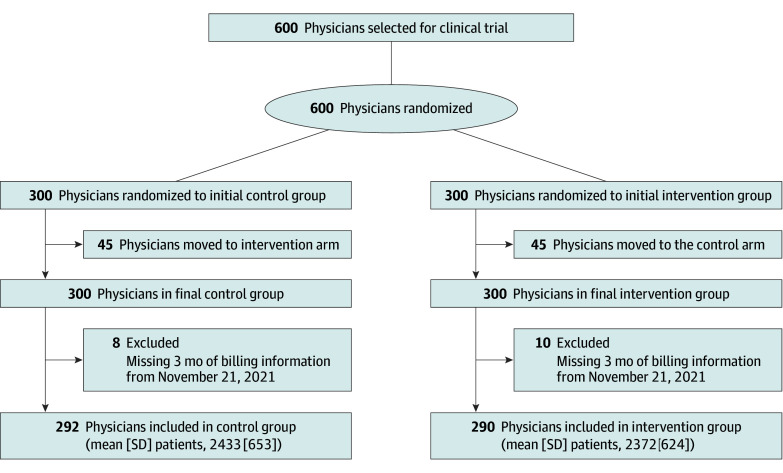
Participant Flow in the Trial of Practice Facilitation for Vaccine Uptake

The trial included a theory-informed process evaluation.^18^ The trial was approved by the Women’s College Hospital Research Ethics Office and received a waiver of consent^19^ due to risk of selection bias.^20^ Instead, we offered opt-out opportunities and a debrief at trial end. We followed the Consolidated Standards of Reporting Trials (CONSORT) reporting guideline with the pragmatic randomized trial extension^21,22^ and cluster extension^23,24^ informed reporting. The impact of the pandemic is documented following the CONSERVE statement.^25^ The original study protocol and statistical analysis plan are available in Supplement 1. While modifications were made to the primary outcome during the study, our research ethics board did not require formal protocol updates for these changes. The primary outcome was updated as indicated in the trial registry. The study was led by Women’s College Hospital with partnerships from Ontario Health, an agency of the provincial government, and ICES (formerly known as the Institute for Clinical Evaluative Sciences). Analyses were conducted at ICES.

### Participants

Approximately 80% of family physicians in Ontario provide comprehensive care in a Patient Enrolment Model (PEM), with a formal roster of patients, and between 15% and 70% of payment is based on age- and sex-adjusted capitation^26^ (eTable 1 in Supplement 2). Since a mechanism existed for identifying patients rostered to their practice, these PEM family physicians were eligible for the COVID-19 vaccine report produced by Ontario Health, a key prerequisite for participation in the study. We determined this aspect of eligibility based on the 6690 family physicians with active Ontario Health digital identity and authentication system (ONE-ID) accounts (approximately 91% of the 7350 accounts on ONE-ID) through eHealth Ontario. This digital identity and authentication system allows health care professionals to securely access electronic health care applications with a single username and password. An independent analyst at Ontario Health then identified the 600 physicians with the largest number of unvaccinated patients. A total sample size of 600 family physicians was identified by our health system decision-maker partners at Ontario Health, who believed that they could not support an intervention for more than 300 family physicians.

### Intervention

In collaboration with Ontario Health, we designed a cluster-randomized clinical trial to evaluate implementation of a mostly telephone- or email-based (and in-person as needed) practice facilitator program to support family physicians in accessing their COVID-19 vaccine report and encouraging COVID-19 vaccine uptake (eAppendix 1 in Supplement 2). Intervention materials were codesigned with family physicians, the research team (J.S., D.K., T.K., and N.I.), and community ambassadors (lay health advisors) through workshops and interviews.

Practices randomized to the intervention arm were contacted by practice facilitators and offered a menu of support services. A detailed list of resources and supports offered to family physicians is available in the Box.^27^ Practices in the control arm did not receive the intervention and continued with standard care.

Box. **Resources and Supports Offered to Family Physicians Through Practice Facilitation in the Intervention Group**

Technical support to access the government vaccine management system (COVaxON) report and merge it with clinic’s electronic medical records.
Written materials for outreach campaign included scripts from administrative staff to call patients and email templates for outreach.
Information regarding where to access robocall and suggested templates.
Trained medical student volunteers to act as delegates to contact patients on the physician’s behalf. These volunteers, who were trained virtually via Zoom in motivational interviewing and the PrOTCT framework,^27^ assisted physicians by conducting patient outreach and motivational interviews.
Communication templates and resources to address common questions and build vaccine confidence including FAQs.
The option to connect patients to trained community ambassadors (lay health advisors working in communities).

Abbreviations: FAQs, frequently asked questions; PrOTCT, presume, offer, tailor, concerns, and talk.

Six practice facilitators received virtual training (Zoom; Zoom Communications Inc) to deliver a 4-month intervention (eAppendix 1 in Supplement 2). An email and fax were sent by Ontario Health to all physicians in the intervention group to offer these supports for contacting unvaccinated patients. Practice facilitators subsequently followed up with weekly phone calls to actively engage physicians and inform them about the supports available to them. Supports were tailored to the specific needs of the clinic or family physician when requested (eg, rural physicians with specialized needs were provided additional resources). Both Ontario Health and the practice facilitators sent multiple faxes and used outreach strategies throughout the 4-month intervention period to engage more physicians. Outreach to the intervention group commenced on November 15, 2021, and continued for 4 months until March 15, 2022.

### Randomization

The randomization process was conducted by staff at Ontario Health where the data were held. Using the list of 600 active physicians who had the largest number of unvaccinated patients, the independent statistician (J.M.) conducted simple randomization by generating a 1:1 allocation sequence using a random number generator in Excel, version 3503 (Microsoft Corporation). Physicians on the list who practiced at the same primary location were then identified by the independent analyst at Ontario Health. To avoid having multiple physicians at the same location allocated to different arms, 45 physicians who were initially randomized to the control arm were moved into the intervention arm. To balance the groups, using a random number sequence, 45 other physicians were selected by the independent statistician from the intervention group to be moved into the control arm.

### Outcomes

The primary outcome was the number of vaccine doses administered during the 4-month follow-up interval (from the time of randomization until follow-up completed at the end of the fiscal year, March 31, 2022) among rostered patients 12 years and older (ie, unit of analysis was the patient). Secondary outcomes were receipt of first, second, and third doses among rostered patients 12 years and older, as well as receipt of any pediatric doses among rostered patients aged 5 to 11 years during the intervention period. Outcomes were measured from the time of randomization until the end of the follow-up period (March 31, 2022) and summarized as vaccine doses per 100 patients.

The primary outcome was originally defined as the proportion of patients eligible for vaccination but not yet vaccinated (ie, overdue) within the participating physician practices at the end of the intervention. However, after the trial began but prior to any analysis, we revised the primary outcome due to significant changes in the public health landscape. The introduction of recommendations for a third COVID-19 vaccine dose, coupled with the emergence of the Omicron variant, necessitated an adjustment to the content of our intervention, which now included encouraging patients to receive the third dose. Therefore, we updated the primary outcome to the number of vaccine doses to better capture the effects of the intervention in relation to the third dose.

### Power Calculation

Trial sample size was predetermined by our ability to deliver the intervention to approximately 300 physicians. Therefore, we selected 600 physicians for randomization, assuming equal allocation. We estimated that the 600 physicians were located at approximately 400 practices (unit of randomization) with an average of 1500 patients per practice. This sample size achieves 88% power to detect a rate ratio of 1.25 using a 2-sided *t* test at the 5% significance level, assuming a control arm vaccination rate of 70 per 100 patients. This would provide power to observe a vaccination rate in the intervention arm of 87.5 or more per 100 patients. This calculation adjusted for clustering using a between-cluster coefficient of variation of 0.70.

### Data Sources

Linked datasets were analyzed at ICES, an independent, nonprofit corporation that analyzes data collected from administering Ontario’s publicly funded universal health care system. These databases have been validated^28^ and used extensively for health services research in Ontario.^29^ Additional information can be found in eAppendix 2 in Supplement 2.

### Baseline Characteristics of Interest

We collected data on sex and years of practice for each family physician. Practice characteristics included roster size and the various primary care enrolment models (enhanced fee-for-service, blended capitation, blended capitation with an interprofessional Family Health Team) and payment via fee-for-service only. Explanation of funding models and Ontario’s PEM are in eTable 1 in Supplement 2. Patient demographic characteristics included age, sex, and overall use of health care resources assessed using the Johns Hopkins Adjusted Clinical Groups.^30^ Resource Utilization Bands over the previous 2 years were calculated, with 0 being no health care use and 10 being the highest expected use. We used Matheson’s Canadian Marginalization Index^31,32^ to assign marginalization quintiles for 2 components of marginalization (1 indicates lowest and 5 indicates highest): dependency (age and labor force) and ethnic concentration (racialized [proportion of people identifying as visible minority groups] and newcomer populations).

### Statistical Analysis

Data were analyzed from March 2023 to May 2024. Baseline characteristics were compared between the intervention and control arms using standardized differences. Standardized differences greater than 0.2 were considered indicative of important differences.

The primary analysis was by intention-to-treat; unit of analysis was the patient. The primary outcome, number of vaccine doses (1, 2, or 3) in the 4 months after randomization, was analyzed using the modified Poisson regression method with robust SEs and an independence correlation structure to account for clustering by practice location.^33^ As odds ratios are commonly misinterpreted and the event of interest was not expected to be rare, we chose to use the modified Poisson regression method rather than logistic regression to estimate the relative risk (RR) reduction directly.^33^ This is a recommended approach for estimating RR, is not prone to convergence problems, and has been validated for its performance in cluster-randomized clinical trials with correlated binary data, provided the total number of clusters is at least 50. The use of the robust sandwich variance estimator protects against misspecification of the correlation structure. We then stratified patients by the number of doses they received. For the first dose analysis, we included patients who did not receive any doses and measured the count of first doses. For the second dose analysis, we included patients who received 1 dose and measured the number of second doses. For the third dose analysis, we included those with 2 doses and measured the number of third doses. We used the same modified Poisson regression method,^33^ and we calculated the rate of doses per 100 patients using the least square means with 95% CIs.

Treatment effects were expressed as RR with 95% CI. We decided a priori to adjust for characteristics known to be associated with COVID-19 vaccination. These variables included physician sex, physician years since graduation, roster size, patient sex, patient age, patient dependency (age and labor force), patient ethnic concentration quintile (racialized and newcomer populations), and patient comorbidity (adjusted clinical group score).^34^

We also conducted a hypothesis-generating receiving-treatment analysis. We compared physician and patient characteristics between practices exposed and unexposed to the intervention using descriptive statistics. The primary outcome, number of vaccine doses (1, 2, or 3) in the 4 months after randomization, was used to analyze using the same Poisson regression method to examine impact of those who were exposed to the intervention compared with controls.

## Results

Of 600 randomized physicians, 18 (3.0%) were excluded during the analysis as nonpracticing family physicians (ie, they did not have any billing for a 3-month period from August 15 to November 15, 2021), 8 from the control arm and 10 from the intervention group. Thus, our analyses included 292 physicians in the control group and 290 in the intervention group (Figure), clustered into 250 practice locations in the control group and 289 practice locations in the intervention group. Of the total 582 physicians, 348 (59.8%) were aged 45 to 64 years (median age, 58 [IQR, 52-66] years); 156 (26.8%) were female and 426 (73.2%) were male (Table 1). Of the 290 physicians in the intervention group, 84 (29.0%) agreed to receive support from the practice facilitator. Of these 84 family physicians, 55 (65.5%) received technical support to identify unvaccinated patients, 23 (27.4%) used trained medical student volunteers to contact patients on their behalf, and 22 (26.2%) used automated calling to reach patients.

**Table 1.** Characteristics at Baseline of Physicians and Their Practices Randomized to Control or Intervention

Characteristic	No./total No. (%)			Standardized difference
	Control group	Intervention group	All	
Physician				
Sex				
Female	74/292 (25.3)	82/290 (28.3)	156/582 (26.8)	0.07
Male	218/292 (74.7)	208/290 (71.7)	426/582 (73.2)	0.07
Training location				
Canada	125/292 (42.8)	136/290 (46.9)	261/582 (44.8)	0.08
Outside Canada, y	21/292 (7.2)	16/290 (5.5)	37/582 (6.4)	0.07
Missing	146/292 (50.00)	138/290 (47.6)	284/582 (48.8)	0.05
Age, median (IQR), y	58 (52-65)	59 (52-66)	58 (52-66)	0.10
Time since graduation, median (IQR), y	32 (25-38)	33 (26-40)	32 (25-39)	0.09
Practice				
Practice type				
CAP	82/292 (28.1)	76/290 (26.2)	158/582 (27.1)	0.04
CCM	18/292 (6.2)	16/290 (5.5)	34/582 (5.8)	0.03
FHG	166/292 (56.8)	150/290 (51.7)	316/582 (54.3)	0.10
FHT	26/292 (8.9)	46/290 (15.9)	72/582 (12.4)	0.21
OGP	0/292	≤5/290 (0.7)	≤5/582 (0.3)	0.12
Rurality				
Urban	256/292 (87.7)	238/290 (82.1)	494/582 (84.9)	0.16
Rural	36/292 (12.3)	52/290 (17.9)	88/582 (15.1)	0.16
No. of rostered patients, median (IQR)	2399 (2024-2747)	2394 (1907-2829)	2395 (1966-2761)	0.06
Patients				
Age, median (IQR), y	44 (26-60)	44 (25-60)	44 (26-60)	0.01
Sex				
Female	310 833/632 818 (49.1)	307 264/619 417 (49.6)	618 097/1 252 235 (49.4)	0.01
Male	321 985/632 818 (50.9)	312 153/619 417 (50.4)	634 138/1 252 235 (50.6)	0.01
New arrival to Ontario				
No	510 517/582 599 (87.6)	519 949/570 879 (91.1)	1 030 466/1 160 825 (89.4)	0.11
Yes	72 082/582 599 (12.4)	50 930/570 879 (8.9)	123 392/1 160 825 (10.6)	0.11
Racialized and newcomer populations, quintile				
1 (Lowest)	46 466/634 434 (7.3)	56 734/618 700 (9.2)	103 200/1 253 134 (8.2)	0.07
2	68 350/634 434 (10.8)	79 358/618 700 (12.8)	147 708/1 253 134 (11.8)	0.06
3	85 606/634 434 (13.5)	91 072/618 700 (14.7)	176 678/1 253 134 (14.1)	0.04
4	137 836/634 434 (21.7)	128 253/618 700 (20.7)	266 089/1 253 134 (21.2)	0.02
5 (Highest)	296 176/634 434 (46.7)	263 283/618 700 (42.6)	559 459/1 253 134 (44.6)	0.08
No. of vaccine doses/100 patients, mean (SD)				
All new doses among patients aged ≥12 y	81.0 (6.3)	79.7 (7.4)	80.4 (6.9)	0.18
First dose among patients aged ≥12 y	80.6 (6.2)	79.4 (7.3)	80.0 (6.8)	0.17
Second dose among patients aged ≥12 y	77.7 (6.9)	76.5 (8.1)	77.1 (7.5)	0.16

Abbreviations: CAP, capitation; CCM, comprehensive care model; FHG, family health group; FHT, family health team; OGP, other primary care model.

### Intention-to-Treat Analysis

The mean number of new doses of COVID-19 vaccines per 100 patients during the intervention period was 49.8 (95% CI, 48.8-50.9) in the intervention group and 50.2 (95% CI, 49.2-51.2) in the control group (adjusted RR [ARR], 0.99; 95% CI, 0.96-1.02) (Table 2). The rate of first doses per 100 patients was 9.0 (95% CI, 8.6-9.4) in the intervention group and 8.8 (95% CI 8.5-9.2) in the control group (ARR, 1.02; 95% CI, 0.97-1.07). The rate of second doses per 100 patients was 3.3 (95% CI 3.1-3.6) in the intervention group and 3.1 (95% CI, 2.9-3.3) in the control group (ARR, 1.08; 95% CI, 0.99-1.17). The rate of third doses per 100 patients was 54.2 (95% CI, 52.9-55.5) in the intervention group and 54.8 (95% CI, 53.6-55.9) in the control group (ARR, 0.99; 95% CI, 0.96-1.02). The rate of pediatric doses per 100 patients was 71.5 (95% CI, 67.6-75.5) in the intervention group and 71.9 (95% CI, 68.5-75.4) in the control group (ARR, 1.01; 95% CI, 1.00-1.02).

**Table 2.** Number of New COVID-19 Doses During the Intervention Period Per 100 Patients by Control and Intervention

Analysis group	Least square mean (95% CI)		ARR (95% CI)	*P* value^a^
	Control group	Intervention group		
No. of physicians	292	290	NA	NA
Primary analysis				
No. of new vaccine doses among patients aged ≥12 y/100 patients	50.2 (49.2-51.2)	49.8 (48.8-50.9)	0.99 (0.96-1.02)	.61
No. of patients	564 793	551 455	NA	NA
Stratified group analysis				
No. of new first vaccine doses among patients aged ≥12 y/100 patients	8.8 (8.5-9.2)	9.0 (8.6-9.4)	1.02 (0.97-1.07)	.48
No. of patients	101 446	104 394	NA	NA
No. of new second vaccine doses among patients aged ≥12 y/100 patients	3.1 (2.9-3.3)	3.3 (3.1-3.6)	1.08 (0.99-1.17)	.10
No. of patients	473 581	457 798	NA	NA
No. of new third vaccine doses among patients aged ≥12 y/100 patients	54.8 (53.6-55.9)	54.2 (52.9-55.5)	0.99 (0.96-1.02)	.51
No. of patients	464 682	449 295	NA	NA
No. of new vaccine doses among patients aged 5-11 y/100 patients	71.9 (68.5-75.4)	71.5 (67.6-75.5)	1.01 (1.00-1.02)	.12
No. of patients	38 288	37 307	NA	NA

Abbreviations: ARR, adjusted relative risk; NA, not applicable.

^a^ Calculated using Poisson count model that accounts for clustering at the physician practice level during 4 months (November 15, 2021, to March 31, 2022), adjusted for physician sex, physician years since graduation, roster size, patient sex, patient age, patient dependency (age and labor force), patient ethnic concentration quintile (racialized and newcomer populations), and patient comorbidity (adjusted clinical group score).

### Receiving-Treatment Analysis

Compared with those who did not engage, physicians who accepted supports were more often female (32 of 84 [38.1%] vs 50 of 206 [24.3%]) and had practices in an urban location (80 of 84 [95.2%] vs 158 of 206 [76.7%]) (Table 3). Their patients were from areas with higher concentrations of people who are racialized and newcomers (52.8% vs 36.7% of patients in quintile 5).

**Table 3.** Characteristics at Baseline of Physicians and Their Practices Among Control, Engaged With Intervention, and Not Engaged With Intervention

Characteristic	No./total No. (%)				*P* value^a^
	Engaged (n = 84)	Did not engage (n = 206)	Control (n = 292)	Total (N = 582)	
Physician					
Sex					
Female	32/84 (38.1)	50/206 (24.3)	74/292 (25.3)	156/582 (26.8)	.04
Male	52/84 (61.9)	156/206 (75.7)	218/292 (74.7)	426/582 (73.2)	
Training					
Canada	34/84 (40.5)	102/206 (49.5)	125/292 (42.8)	261/582 (44.8)	.50
Outside Canada	5/84 (6.0)	11/206 (5.3)	21/292 (7.2)	37/582 (6.4)	
Missing	45/84 (53.6)	93/206 (45.1)	146/292 (50.00)	284/582 (48.8)	
Time since graduation, median (IQR), y	32 (25-37)	34 (26-40)	32 (25-38)	32 (25-39)	.17
Practice					
Practice type					
CAP	27/84 (32.1)	49/206 (23.8)	82/292 (28.1)	158/582 (27.1)	.13
CCM	5/84 (6.0)	11/206 (5.3)	18/292 (6.2)	34/582 (5.8)	
FHG	39/84 (46.4)	111/206 (53.9)	166/292 (56.8)	316/582 (54.3)	
FHT	13/84 (15.5)	33/206 (16.0)	26/292 (8.9)	72/582 (12.4)	
OGP	0/84	<5/206 (1.0)	0/292	≤5/582 (0.3)	
Rurality					
Urban	80/84 (95.2)	158/206 (76.7)	256/292 (87.7)	494/582 (84.9)	<.001
Rural	<5/84 (4.8)	48/206 (23.3)	36/292 (12.3)	88/582 (15.1)	
No. of rostered patients, median (IQR)	2422 (1894-2934)	2372 (1912-2729)	2389 (2010-2740)	2386 (1953-2752)	.71
Patient					
Age, median (IQR), y	43 (25-59)	44 (26-61)	44 (26-60)	44 (26-60)	<.001
Sex					
Female	90 688/182 034 (49.8)	216 576/437 383 (49.5)	310 833/632 818 (49.1)	618 097/1 252 235 (49.4)	<.001
Male	91 346/182 034 (50.2)	220 807/437 383 (50.5)	321 985/632 818 (50.9)	634 138/1 253 235 (50.6)	
New arrival to Ontario					
No	147 971/166 992 (88.6)	371 978/403 887 (92.1)	510 517/582 599 (87.6)	1 030 466/1 153 478 (89.3)	<.001
Yes	19 021/166 992 (11.4)	31 909/403 887 (7.9)	72 082/582 599 (12.4)	123 012/1 153 478 (10.7)	
Racialized and newcomer populations, quintile					
1 (Lowest)	8947/182 034 (4.9)	52 109/437 383 (11.9)	47 283/632 818 (7.5)	108 339/1 252 235 (8.7)	<.001
2	14 272/182 034 (7.8)	65 988/437 383 (15.1)	62 981/632 818 (10.0)	143 241/1 252 235 (11.4)	
3	21 563/182 034 (11.8)	66 249/437 383 (15.1)	86 393/632 818 (13.7)	174 205/1 252 235 (13.9)	
4	41 221/182 034 (22.6)	92 687/437 383 (21.2)	147 204/632 818 (23.3)	281 112/1 252 235 (22.4)	
5 (Highest)	96 031/182 034 (52.8)	160 350/437 383 (36.7)	288 957/632 818 (45.7)	545 338/1 252 235 (43.5)	
No. of vaccine doses/100 patients, mean (SD)					
All new doses among patients aged ≥12 y	81.8 (6.3)	81.3 (6.7)	82.4 (5.7)	81.9 (6.2)	.14
First dose among patients aged ≥12 y	81.7 (6.3)	81.3 (6.7)	82.4 (5.7)	81.9 (6.2)	.14
Second dose among patients aged ≥12 y	80.0 (6.8)	79.5 (7.1)	80.6 (6.15)	80.1 (6.6)	.18

Abbreviations: CAP, capitation; CCM, comprehensive care model; FHG, family health group; FHT, family health team; OGP, other primary care model.

^a^ Calculated using 3-way χ^2^ analysis.

There were no differences for primary or secondary outcomes when comparing vaccine uptake among those who engaged with the intervention supports and those who did not (eTable 2 in Supplement 2). The mean number of doses of COVID-19 vaccines per 100 patients during the intervention period among those who engaged was 50.4 (95% CI, 48.3-52.5) compared with 50.2 (95% CI, 49.2-51.2) in the control group (ARR, 1.00; 95% CI, 0.96-1.05). The mean rate of first doses per 100 patients was 8.8 (95% CI, 8.2-9.4) among those who engaged and 8.8 (95% CI 8.5-9.2) in the control group (ARR, 1.02; 95% CI, 0.95-1.10). The mean rate of second doses per 100 patients was 3.3 (95% CI, 2.9-3.7) among those who engaged and 3.1 (95% CI, 2.9-3.3) in the control group (ARR, 1.06; 95% CI, 0.93-1.20). The mean rate of third doses per 100 patients was 54.7 (95% CI, 52.3-57.3) among those who engaged and 54.8 (95% CI, 53.6-55.9) in the control group (ARR, 1.00; 95% CI, 0.95-1.05). The mean rate of pediatric doses per 100 patients was 73.1 (95% CI, 65.9-81.1) among those who engaged and 71.9 (95% CI, 68.5-75.4) in the control group (ARR, 1.01; 95% CI, 1.00-1.03).

## Discussion

This cluster-randomized clinical trial of practice facilitation for primary care physicians represented a unique partnership between a government agency and an academic research team. We did not find a difference in the uptake of COVID-19 vaccinations in the intention-to-treat analysis or in the receiving-treatment analysis, although such analyses were underpowered.

While most interventions for increasing COVID-19 vaccine uptake target the individual directly,^35^ this intervention tested the idea that we could build on the existing strengths of the family physician as a trusted messenger to advocate for vaccination (eg, through disseminating materials or clinical encounters), provide fact-based responses to individual concerns, and redirect individuals to care if needed. A prior randomized clinical trial^36^ found that primary care outreach using electronic and mailed messages increased COVID-19 vaccination rates among Black and Latino older adults.

The timing overlapped with the onset of the Omicron variant wave, when health services in Ontario were stretched to their limit. COVID-19 vaccines were already readily available to all Ontarians for 6 months, and 86% of individuals 12 years and older were fully vaccinated.^37^ There were also considerable efforts by public health units to increase vaccine uptake. It is possible that by the time the intervention started, patients who had not already been vaccinated may not have been amenable to discussion with their family physician. Physicians at this time were also facing high levels of burnout.

Notably, few family physicians had COVID-19 vaccines available in their practices to offer patients, which placed the onus on the patient to coordinate logistics. A systematic review^35^ found that personalizing communications and sending booking reminders via text message increased vaccine uptake, suggesting that individual conversations are necessary but may be insufficient without the ability to book a vaccination appointment in parallel.

Only 29.0% of physicians in the intervention group accepted help from the practice facilitators. The lower-than-anticipated engagement rate made detecting differences between intervention and control groups difficult. Also, our analysis did not capture the fidelity of the intervention. For example, we did not capture whether those who accepted technical support to identify unvaccinated patients acted after they were identified or if those who received communication materials distributed materials in their clinic. We found that physicians who accepted supports were more likely to be in urban locations, and few physicians practicing in rural areas accepted additional support. Tailoring programs specifically for physicians in rural and remote regions might increase future participation and highlight the need to tailor interventions according to unique contexts.

A unique feature of this trial was that participants were selected based on data alone; physicians with the largest number of unvaccinated patients were randomized (without consent) to receive outreach. This was enacted purposefully to understand how data-informed outreach might work and allowed us to estimate effects most in keeping with how such a program might be implemented in the future, involving targeted support of specific practices. Testing this question required alteration to the usual approach to consent, as randomizing only consenting practices would have biased the study sample and indeed changed the study question.^38^ We believe that if the intervention was broadly advertised, without targeted outreach, the intervention could exacerbate inequalities by providing additional resources to those with greater capacity and resources.^39^ Ideally, when targeting recipients for an intervention, there should be room for improvement, but a readiness for change for the individual and organization is required.

### Limitations

There were several limitations to implementing the trial that should be considered. First, the results may not be generalizable beyond this unique period or extended to non–COVID-19 vaccines. Second, analysis of patient and physician characteristics was limited to administrative databases. Therefore, we were unable to measure larger social determinants of health, psychological factors, vaccination beliefs, and physician burnout, which are all known to be associated with vaccinations. The use of administrative data for patient demographic characteristics and marginalization indices is for the pragmatic nature of the trial; however, it may introduce measurement errors, such as reliance on proxy variables for socioeconomic factors, which could impact the accuracy and completeness of the data. Regarding randomization, ideally, physicians would have been grouped by practice and then randomized. However, the process retained the qualities of random selection. It was possible to create a bias where larger practices are overrepresented in the intervention group; however, practice sizes were not significantly different. An imbalance in the number of physicians between the control and intervention groups, with more exclusions from the control group after randomization, may have reduced statistical power and increased the likelihood of a type II error, potentially contributing to the null findings. It is also important to note that our intervention was limited to patients who were attached to a family physician. Patients with no attachment to primary care likely represent the most significant public health priority during the pandemic and beyond, and future work should examine this group.^6,40,41,42^ Last, the power calculation was based on vaccination rates that did not fully account for the high baseline vaccination rate of 86% during the study period, which may have resulted in an underestimation of the sample size needed to detect a meaningful effect.

## Conclusions

In this randomized clinical trial of practice facilitation with primary care, the intervention did not increase COVID-19 vaccination uptake. Intervention fidelity is a possible explanation for the trial’s null results. Our findings highlight the importance of considering the target population for intervention and a population that is both receptive and has room for improvement. Furthermore, since vaccine counselling is thought to be more effective when conversations and vaccine administration are paired, the findings of this trial emphasize the importance of coordinating public health and primary care efforts in pursuit of population health.

## Notes

Supplement 1.
Trial Protocol

Supplement 2. **eTable 1.** Explanation of Physician Payment Models and Ontario’s Patient Enrollment Model **eAppendix 1.** Training Information for the Practice Facilitators **eAppendix 2.** Administrative Databases **eTable 2.** Number of New COVID-19 Doses During the Intervention Period per 100 Patients, by Control and Engaged

Supplement 3.
Data Sharing Statement
