# Supplementary material for: Practice Facilitation to Support Primary Care Physicians With COVID-19 Vaccine Uptake: A Randomized Clinical Trial
Source: JAMA Netw Open. 2025 May 14;8(5):e259967. doi: 10.1001/jamanetworkopen.2025.9967 (PMC12079287; doi:10.1001/jamanetworkopen.2025.9967)
Supplement: Supplement 1. — Trial Protocol [file jamanetwopen-e259967-s001.pdf]

INTERVENTION DESCRIPTION

Our goal is to help family physicians to effectively engage with those patients that are most in need of their support. Our intervention uses practice facilitators[1-3] to empower family physicians to access their report and use it to help their patients get vaccinated. These practice facilitators use participatory methods and have expertise as adult educators and facilitators of change. They will offer the physician a facilitation session plus an opportunity to participate in a townhall with their patients.

Our practice facilitators will help physicians by:

1. Providing technical support to access the report, activating a delegate on the system, to provide the list with the complete patient roster generated by the electronic medical records which identifies patients that still need to be vaccinated.
2. Helping develop an outreach plan with their administrative staff. Outreach can include bulk emails, text messages, phone call, or an invitation to a townhall. They will provide advice and resources for family physicians and their staff in responding to vaccine-related questions. Every eligible physician (i.e., those with access to a vaccine list) in a given practice location will be able to benefit from the support of the practice facilitator.
3. The physicians will also be offered the help of a medical student volunteer to assist with patient engagement. Under the Health Insurance Portability and Accountability Act (HIPAA), Physicians, as the acting health information custodian, can legally assign a custodian to do something on their behalf. The medical student volunteer will enter into a non-disclosure agreement (NDA) to ensure the patient's privacy and confidentiality of vaccination status. The medical student volunteer can help with patient engagement and offer support to family physicians who might not have the time to personally contact those patients who have yet to receive both doses of the COVID-19 vaccine. The medical student volunteers will be provided with a script for making telephone calls and training in Private Health Information, refraining from providing medical advice and the importance of confidentiality. They will also be provided with a list of common questions to address when contacting patients regarding COVID-19 vaccination and resources on how to engage in conversation with individuals who are vaccine hesitant.. A few topics provided to the medical student volunteer prior to contacting patients will include making the case for vaccination, finding out patient concerns, how to counter common vaccine concerns (speed of vaccine development, safety, side effects, misinformation, distrust of science, government and medical community, underlying conditions that can increase vulnerability to vaccine side effects) and why vaccination matters. According to the Canadian Medical Protective Association (CMPA), the medical student will be covered under the insurance of the physician as long as the physician provides permission to contact the patient. The medical student must also record the interaction using the call log datasheet provided by the research team and send this information back to the Family Physicians to be included in their EMR. A call log form

will be provided for every volunteer to track their calls and the patients who were contacted.

4. Physicians will also be invited to co-host in a townhall with their unvaccinated patients. Each physician will have the opportunity to organize one town hall for their patients. The facilitator and our research staff will support these town halls, including a template slide deck, and customizable promotional material, all developed in partnership with the Health Commons Solutions Lab. Family physicians will take part in the session so they can address any clinical questions. The family physician will be offered a stipend for their time involved in inviting unvaccinated patients to the sessions and participation. Following the webinar, family physicians will be supported to send targeted letters to each of their as-yet-unvaccinated patients with action plans and supportive materials. Physicians working in communities with community ambassadors (i.e. a lay health advisor) will also have opportunity to include a community ambassador in their town hall. Community ambassadors can co-host the townhall and play an integral piece as they have connections with the community and understand the context and their struggles.

RESEARCH QUESTIONS:

Our questions are informed by both an equity lens and the RE-AIM framework[4-6]:

- **Reach:** In how many practices did the intervention occur and how did those practices overlap geographically with maps of COVID-19 impact and vaccine uptake?

- **Effectiveness:** Does the intervention increase vaccine uptake overall and does it help reduce inequities in vaccine uptake compared to the audit and feedback intervention alone? In which types of family physicians and for which types of patients did the interventions work best?

- **Adoption:** How many family physicians, primary care practices, community ambassadors, and patients engaged, from which communities, and why?

- **Implementation:** What are the factors that facilitated or hindered implementation and by what mechanisms may the intervention help with vaccine uptake in different communities? How did family physicians, community ambassadors, and patients perceive the effects of the initiative?

- **Maintenance:** How do family physicians, practice facilitators, and community ambassadors envision ways to sustain and scale the initiative? What other applications in the health promotion and illness prevention space could benefit from this approach?

EVALUATION

Study Design: We will conduct a two-arm, pragmatic[7] (Figure 1), cluster randomized trial, with an embedded, theory-informed process evaluation and economic evaluation, building on our recent guidance for best practices in implementation trials[8]. We chose this design because we wanted a rapid yet rigorous evaluation, and our partners wanted to ensure that all physicians had a chance to receive the intervention.

Population: Our population consists of 6,690 family physicians across Ontario who have active (up-to-date passwords) ONE ID accounts (this is approximately 91% of the 7350 accounts which are available for use).

Allocation: An independent statistician at Ontario Health will use internal data from COVaxON registry data to identify the top 600 physicians who have the largest number of unvaccinated patients. Physicians that were in the same practice were then identified. A randomization sequence was then applied separating the physicians 1:1 to either the intervention or control group. Physicians in the control group who practiced at the same location at physicians in the control group will be moved into the intervention group and an equal number will be randomly selected to move to the control group to equalize the groups. This will limit contamination as all physicians at the same location will wither be in the control or intervention group. The trial wouldn't be feasible without a waiver of consent[9] since explicit consent would lead to selection bias[10]. Instead, we will offer opt-out opportunities, and a debrief at trial-end.

Recruitment to Intervention: Ontario Health will send out emails and faxes to physicians in the intervention group that will explain to them that they have a large group of eligible and unvaccinated patients and that an initiative is planned to support them in reaching out to those patients, with an embedded evaluation. It will ask them to reach out to the research team to plan a time to access the supports to gather more information, or to opt-out from the evaluation. Specifically, physicians will receive invitations to receive practice facilitation via mail letter and fax, followed by up to five weekly phone calls from a team member at Ontario Health. We will send out multiple emails to ensure that the physician has received the letter of support.

Trial Data Collection and Outcomes: The primary outcome is the proportion of patients eligible-but-not-yet vaccinated (overdue) within participating physician practices after 4 months. We will analyze this overall and specifically among individuals from communities with a high marginalization index (an empirically derived, theoretically informed tool that reflects four dimensions of marginalization: residential instability, material deprivation, dependency and ethnic concentration) [11]. This index is necessary because patient-level ethnicity and race-based data are not available at the population level and disparities in COVID-19 vaccination have been observed already at the small-area level. We will assess characteristics of clinics (e.g., rurality, size of clinic), physicians (e.g., years in practice, sex, training in Canada, number of

patients, practice model and billings), and patients (e.g., comorbidity index, immigration status, neighbourhood marginalization index, age, sex).

Data analysis will take place at ICES. Ontario Health will provide ICES with a list of physicians that were in either the control or intervention group. This list will be transferred to ICES using a secure portal process (axway). Data will be captured from administrative databases (including COVaxON registry data) held at ICES. Data will be linked at the physician level using their encrypted CPSO number. This team has done this type of linkage in previous projects and has received privacy reviews. Additional ICES databases will be linked: ICES Physician Database, Ontario Marginalization Index, Primary Care Population, and the Client Agency Program Enrolment, Discharge Abstract Database, National Ambulatory Care Reporting System, IRCC Permanent Residents database, GAPP Decision Support Systems (Physician Payments), Postal Code Conversion File, COVID19 Integrated Testing Data, and Registered Persons Database.

ICES is an independent, non-profit research institute whose legal status under Ontario's health information privacy law allows it to collect and analyze health care and demographic data, without consent, for health system evaluation and improvement. ICES privacy policy and safeguards will be followed to ensure no patient identifiers are used and data at the physician level with <6 cells will be reported as <6. All parties involved will agree to an information sharing agreement prior to any information exchange.

Trial Analysis: Analyses will be conducted at the level of the patient and will follow the intent-to-treat principle. We will describe characteristics of clinics, physicians, and patients for each of the randomized arms. We will compare the proportion of patients 'overdue' between the study arms three months after randomization, using the modified Poisson regression method with robust standard errors and an exchangeable correlation [12] to produce treatment effects estimated as relative risk (RR) and 95% Confidence Interval (CI). We will examine treatment effects within subgroups defined by sex of physician, and by sex of the patient by including these indicators and their interaction with trial arm into the model. We will explore whether treatment effects varied across subgroups of higher and lower numbers of patients that are overdue for vaccines and by proxy measures of equity such as neighbourhood income quintile and marginalization index. We will include these terms in the model with their treatment interaction and report RR with 95% CI within each subgroup. Likewise, we will look at effects for patient-age subgroups: >12 (if eligible during study timeframe) 12-17, 18-25, 26-39, 40-64, 65-79, 80+.

Trial sample size is pre-determined by our resources to deliver the intervention. Approximately 6,690 family physicians across Ontario have active ONE ID accounts. (This is approximately 91% of the 7350 accounts which are available for use.) We will identify the bottom decile of physicians, of those who have the highest number of eligible patients unvaccinated (N≈ 669) and half of these physicians will be randomized to receive the intervention (N=335 physicians). We estimate that this will involve about XXX primary care practices (approximately 3-4 physicians per practice location, on average).

Based on current vaccination rates, we estimate that 20% of patients will be 'overdue' at the trial onset. With an intra-cluster correlation of 0.1, we will have 90% power to detect a minimum further increase in vaccine uptake of 1.5% in the intervention arm, i.e., 20% overdue in the audit and feedback arm versus 18.5% in the intervention arm. Because of the large available sample size, we will also have ~90% power to detect small differences even in the most marginalized quintile.

Finally, to evaluate effects of the audit and feedback alone, we will conduct two sets of analyses. First, we will repeat the above robust Poisson regression analyses by comparing the vaccine uptake between those that did or did not view the report, by study arm. This model will adjust for clinic, physician, and patient characteristics that may be associated with audit and feedback exposure. Next, we will conduct Cox proportional hazards regression analyses with robust standard errors to account for clustering, by identifying a cohort of patients who are unvaccinated on September 1, 2021 and following them until they are vaccinated or until the end of the study, with the time at which their physician receives the audit and feedback defined as a time-dependent variable.

Process Evaluation: Our process evaluation[13] will allow us to explore important insights into the implementation of the intervention, mechanisms of change, and contextual factors which can help inform how to optimize, and how to spread and scale the intervention to new contexts. We will describe the proportion of all Ontario family physicians sent a feedback report and/or a intervention (and the type of practice model they work within), the total proportion of 'overdue' patients, and the proportion of overdue patients in areas with a high marginalization index potentially reached through the trial will be compared to the overall Ontario population. We will describe the number of physicians that we supported through town halls (and the number of sessions with a community ambassador) and number of attendees. Participants will be invited to answer a 5-minute survey at the end of the session regarding their perception of the program and vaccination intentions.

Fidelity of receipt[14] will be assessed by identifying the proportion of eligible intervention group physicians that accessed the interventions. For the online audit and feedback reports, we will be able to track which physicians accessed and how often using website log-in data.

Fidelity of enactment[15] will be explored through telephone interviews with family physicians to identify barriers and enablers to engaging with both the Ontario Health report and the intervention. Leveraging our knowledge users and collaborators, we will aim to recruit at least 30 physicians (seeking equal groups of those who did and did not use engage with the intervention). We will offer a $150 honorarium with an Amazon e-gift card, to encourage participation. Recruitment will take place by sending invitation letters to physicians who received the intervention letter from Ontario Health. Invitation letters will come from Ontario Health with a request to contact the research team. We will supplement this with recruitment through social media and networks of the investigator team. A member of the research team

will contact all those who respond to the invitation, explain the study, review the study information and obtain consent if they wish to participate.

Interviews with physicians will explore why they engaged and their perceptions of the program. Interviews will be informed by the Theoretical Domains Framework, a comprehensive, theory-informed approach often used in implementation research to understand determinants of targeted behaviours[16]. Each behaviour related to the desired intervention processes (e.g., accessing the report, discussing aspects of toolkit with clinic staff, contacting patients) will be specified[17] and explored to understand barriers and enablers and inform future intervention strategies[18]. For example, we will explore whether, why, and how beliefs in capabilities (i.e., confidence) in effectively carrying out vaccine outreach was changed by the toolkit and how that relates to beliefs in capabilities in those who do not receive it. In addition to the Theoretical Domains Framework, we will use the Social Determinant of Health framework to consider the broader determinants of health as part of a more comprehensive approach to addressing vaccine hesitancy [19,20].

The interview guide will be piloted with two physicians using the "think aloud method"[21] and will be revised as needed. Interviews will last approximately 30-45 minutes and will be conducted by telephone or Zoom and recorded for transcription, coding and analysis. Brief demographic questions will be asked at the beginning of the interview, including: physician sex, years in practice, type of practice, location of practice, and average number of patients seen a day. Remuneration will be sent as an Amazon e-gift card by e-mail after completion of the interview. No subject identifiers will be included in the analysis and presentation of the qualitative findings (all identifying features will be removed from transcription prior to qualitative analysis). Our research will not impact the physician's employment, relationship with provincial organizations or reputation in any way and is meant to be informative for the research community to contribute to the growing and relevant body of research.

We will also conduct interviews with practice facilitators, community ambassadors, and patients who attended the town halls. They will receive multiple invitation letters from the research staff either via mail or fax. If they respond that the are interested in participating a member of the research team will contact them, explain them the study, review the study information, obtain consent and schedule an interview if they wish to participate. Interviews will last approximately 30-45 minutes and will be conducted by telephone or Zoom and recorded for transcription, coding and analysis. Brief demographic questions will be asked at the beginning of the interview, including gender, age, postal code, income, general health state, and vaccination status. Patient, staff and community ambassador interview participants will be offered a 35$ Amazon e-gift card, sent via e-mail as honorarium. We will aim to recruit 20 patients who participated in town halls, 10 community ambassadors and 5 practice facilitators.

The facilitator, community ambassador, and patient interviews will be guided by the model developed by our co-investigators [22] which uses the Capability, Opportunity, and Motivation (COM-B) model[23] and Theoretical Domains Framework[16] to help identify factors affecting

vaccination acceptance and uptake (Figure 2). These factors can be linked to solutions - specific behavior change techniques that can be used to address barriers and enablers to vaccination. This model will allow us to explore different factors of the intervention that may influence vaccine acceptance. Interviews will be recorded and transcribed.

For all qualitative analysis, thematic analysis[24], using inductive and deductive coding with Nvivo, will be used to allow the framework model domains to be integral to analysis while allowing for themes to emerge directly from the data coding. No subject identifiers will be included in the analysis and presentation of the qualitative findings (all identifying features will be removed from transcription prior to qualitative analysis).

Figure 2: Potential drivers of vaccination acceptance and uptake based on the COM-B model and Theoretical Domains Framework, embedded within social and structural factors[22]

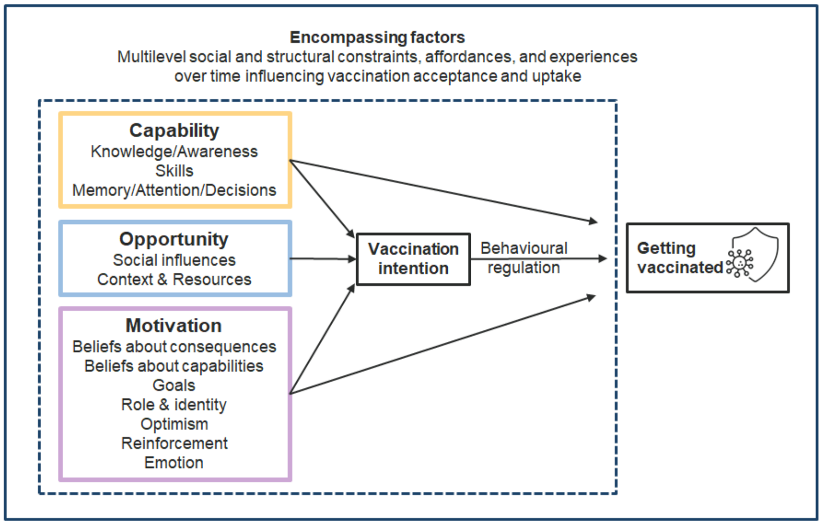

Confidentiality

Interviews will be audio recorded and then transcribed verbatim by an external third party. Any identifiable information (i.e., names of individuals or institutions) will be removed from transcripts to ensure that respondents remain anonymous. Identifiable information will not be used in any project records, except the consent form (which will be stored separately from the other project records). The interview transcripts will be assigned a unique identification code and will be referred by this code during discussions and documents. The audio recordings will be stored in a secure location and viewed only by members of the research team. The recordings will be kept until they have been transcribed (turned into written records), and then they will be destroyed. The transcripts will be kept in a secure location for ten years and then destroyed.

All project files will be stored on the secure internal WCH drive and will only be accessible to personnel listed on the approved REB submission.

Economic evaluation: While interventions specifically to support COVID-19 vaccination may or may not need to be sustained, the same approach could be adapted for future public health issues. A key element to inform the potential sustainability of the intervention is to evaluate the economic return on investment. We will therefore assess the cost-effectiveness of improved vaccine uptake among adults in Ontario. Total costs of delivering the standard (OH report) and intervention (letter with information on supports and town halls) will be identified, measured and valued in collaboration with our partners. Will we draw healthcare costs from ICES. The analysis will be conducted in accordance with current guidelines for clinical and cost-effectiveness analysis alongside randomized control trials[25] and the best practices for the conducting and reporting of health economic evaluations[26]. The costs and effects of the interventions will be compared. We will measure the cost-effectiveness of each arm separately. If either of the interventions are associated with the higher cost and increased effectiveness, the incremental cost per unit change in vaccine uptake will be estimated. Uncertainty in the analysis will be addressed by estimating 95% CIs using a non-parametric bootstrapping method. Acceptability curves will depict the probabilities that the interventions are cost-effective.

References

1. Baskerville NB, Liddy C, Hogg W: Systematic review and meta-analysis of practice facilitation within primary care settings. The Annals of Family Medicine 10:63-74, 2012
2. Agency for Healthcare Research and Quality: Engaging Primary Care Practices in Quality Improvement: Strategies for Practice Facilitators, 2015
3. Higgins T, Schottenfeld L, Crossen J: Primary Care Practice Facilitation Curriculum (Module 25). 2015
4. Glasgow RE, Vogt TM, Boles SM: Evaluating the public health impact of health promotion interventions: the RE-AIM framework. American journal of public health 89:1322-1327, 1999
5. Shelton RC, Chambers DA, Glasgow RE: An extension of RE-AIM to enhance sustainability: addressing dynamic context and promoting health equity over time. Frontiers in Public Health 8:134, 2020
6. Glasgow RE, Askew S, Purcell P, et al: Use of RE-AIM to address health inequities: Application in a low-income community health center-based weight loss and hypertension self-management program. Translational behavioral medicine 3:200-210, 2013
7. Loudon K, Treweek S, Sullivan F, et al: The PRECIS-2 tool: designing trials that are fit for purpose. bmj 350, 2015
8. Wolfenden L, Foy R, Presseau J, et al: Designing and undertaking randomised implementation trials: guide for researchers. bmj 372, 2021
9. Weijer C, Grimshaw JM, Eccles MP, et al: The Ottawa statement on the ethical design and conduct of cluster randomized trials. PLoS Med 9:e1001346, 2012
10. Canadian Institutes of Health Research NSaERCoC, and Social Sciences and Humanities Research Council of Canada T-CPS: Ethical Conduct for Research Involving Humans, December 2014
11. Matheson FI, Dunn JR, Smith KL, et al: Development of the Canadian Marginalization Index: a new tool for the study of inequality. Canadian Journal of Public Health/Revue Canadienne de Sante'e Publique:S12-S16, 2012
12. Zou G, Donner A: Extension of the modified Poisson regression model to prospective studies with correlated binary data. Statistical methods in medical research 22:661-670, 2013
13. Moore GF, Audrey S, Barker M, et al: Process evaluation of complex interventions: Medical Research Council guidance. bmj 350, 2015
14. Rixon L, Baron J, McGale N, et al: Methods used to address fidelity of receipt in health intervention research: a citation analysis and systematic review. BMC Health Services Research 16:1-24, 2016
15. Toomey E, Hardeman W, Hankonen N, et al: Focusing on fidelity: narrative review and recommendations for improving intervention fidelity within trials of health behaviour change interventions. Health psychology and behavioral medicine 8:132-151, 2020
16. Atkins L, Francis J, Islam R, et al: A guide to using the Theoretical Domains Framework of behaviour change to investigate implementation problems. Implementation Science 12:1-18, 2017
17. Presseau J, McCleary N, Lorencatto F, et al: Action, actor, context, target, time (AACTT): a framework for specifying behaviour. Implementation Science 14:1-13, 2019
18. Desveaux L, Saragosa M, Russell K, et al: How and why a multifaceted intervention to improve adherence post-MI worked for some (and could work better for others): an outcome-driven qualitative process evaluation. BMJ open 10:e036750, 2020
19. Solar O, Irwin A: A conceptual framework for action on the social determinants of health, WHO Document Production Services, 2010
20. Dean HD, Williams KM, Fenton KA: From theory to action: applying social determinants of health to public health practice, SAGE Publications Sage CA: Los Angeles, CA, 2013

21. Ramey J, Boren T, Cuddihy E, et al: Does think aloud work? how do we know? Presented at the CHI '06 Extended Abstracts on Human Factors in Computing Systems, Montréal, Québec, Canada, 2006

22. Crawshaw J KK, Castillo G, van Allen Z, Grimshaw JM, Presseau J: Factors affecting COVID-19 vaccination acceptance and uptake among the general public: a living behavioural science evidence synthesis (v2, May 31st, 2021). 2021

23. Michie S, Van Stralen MM, West R: The behaviour change wheel: a new method for characterising and designing behaviour change interventions. Implementation science 6:1-12, 2011

24. Braun V, Clarke V: Using thematic analysis in psychology. Qualitative research in psychology 3:77-101, 2006

25. Glick HA, Doshi JA, Sonnad SS, et al: Economic evaluation in clinical trials, OUP Oxford, 2014

26. Husereau D, Drummond M, Petrou S, et al: Consolidated health economic evaluation reporting standards (CHEERS)—explanation and elaboration: a report of the ISPOR health economic evaluation publication guidelines good reporting practices task force. Value in health 16:231-250, 2013
