# Supplementary material for: Practice Facilitation to Support Primary Care Physicians With COVID-19 Vaccine Uptake: A Randomized Clinical Trial
Source: JAMA Netw Open. 2025 May 14;8(5):e259967. doi: 10.1001/jamanetworkopen.2025.9967 (PMC12079287; doi:10.1001/jamanetworkopen.2025.9967)
Supplement: Supplement 2. — eTable 1. Explanation of Physician Payment Models and Ontario’s Patient Enrollment Model eAppendix 1. Training Information for the Practice Facilitators eAppendix 2. Administrative Databases eTable 2. Number of New COVID-19 Doses During the Intervention Period per 100 Patients, by Control and Engaged [file jamanetwopen-e259967-s002.pdf]

Supplementary Online Content

Shuldiner J, Shah N-U-H, Bar-Ziv S, et al. Practice facilitation to support primary care physicians with COVID-19 vaccine uptake: a randomized clinical trial. *JAMA Netw Open.* 2025;8(5):e259967. doi:10.1001/jamanetworkopen.2025.9967

eTable 1. Explanation of Physician Payment Models and Ontario's Patient Enrollment Model

eAppendix 1. Training Information for the Practice Facilitators

eAppendix 2. Administrative Databases

eTable 2. Number of New COVID-19 Doses During the Intervention Period per 100 Patients, by Control and Engaged

This supplementary material has been provided by the authors to give readers additional information about their work.

© 2025 Shuldiner J et al. *JAMA Network Open*.

eTable 1. Explanation of Physician Payment Models and Ontario's Patient Enrollment Model

Name	Explanation
Comprehensive Care Model	Model for solo physicians. This group includes physicians funded through both entirely or majority fee-for-service. Incentives, premiums and bonuses, chronic disease management and preventive care are paid for eligible services to enrolled patients.
Capitation	These models require patient rostering. The main example is Family Health Organizations - physicians in these models are paid mainly via capitation blended with relatively small amounts by fee-for-service, plus minor incentive and bonus components, with certain requirements for after hours care.
Family Health Team	Physicians in these models are usually paid as per the capitation group, but also benefit from formal links to government-funded interdisciplinary teams (e.g. registered nurses, dietitians, social workers, pharmacists) providing ongoing health care.
Family Health Group	Model for at least 3 physicians. Majority of income through fee for service billings. Incentives, premiums and bonuses, chronic disease management and preventive care are paid for eligible services to enrolled patients.
Other primary care models	These models are mainly salary-based, such as those working in Community Health Centers, which employ teams of physicians, nurse practitioners, nurses, counsellors, community workers and dieticians that serve high-risk communities and populations who may have trouble accessing health services because of language, culture, physical disabilities, socioeconomic status or geographic isolation.

© 2025 Shuldiner J et al. *JAMA Network Open*.

eAppendix 1. Training Information for the Practice Facilitators

Six practice facilitators were virtually trained on Zoom to deliver a four-month intervention. Practice facilitators were trained in: facilitation techniques, including modules on communication and recruitment skills through Health Innovation Group (https://healthinnovationgroup.ca); primary care clinic management in Ontario (e.g., workflow, operations, structure); COVID-19 vaccine myths and facts through research team members; and technical aspects of the commonly used primary care EMR software (e.g. Telus PSS, Accuro, OSCAR), including how they can be linked to the Ontario Health report to identify patients who have not been vaccinated (supported by OntarioMD[27]).

© 2025 Shuldiner J et al. *JAMA Network Open*.

eAppendix 2. Administrative Databases

We measured vaccine uptake by linking the provincial vaccine registry to routinely used administrative databases (including COVaxON registry data, ICES Physician Database, Ontario Marginalization Index (ON-Marg), Primary Care Population, and the Client Agency Program Enrolment, Discharge Abstract Database, National Ambulatory Care Reporting System, IRCC Permanent Residents database, GAPP Decision Support Systems (Physician Payments), Postal Code Conversion File, COVID19 Integrated Testing Data, and Registered Persons Database) from ICES. Data was linked at the physician level using their encrypted College of Physicians and Surgeons of Ontario number, a unique physician identifier. Secondary analysis used geographic area-level marginalization indexes: dependency, and ethnic concentration[11]. ICES data sets have been shown to be valid for socio-demographic data[1], physician billing claims[1], permanent resident status[28,29] and Covid-19 vaccinations[30].

© 2025 Shuldiner J et al. *JAMA Network Open*.

eTable 2. Number of New COVID-19 Doses During the Intervention Period per 100 Patients, by Control and Engaged

	**Control**<br><br>**N=293**<br>LS mean (95% CI)	**Engage**<br><br>**N=84**<br>LS mean (95% CI)	**Adjusted Relative Rate**<br>**95% CI, p value***
**Primary analyses**			
Count of vaccine doses: patients 12+/100 patients	50.2 (49.2-51.2)<br>N patients=564,793	50.4 (48.3-52.5)<br>N patients= 160,356	1.00 (0.96-1.05), P=0.45
**Stratified analyses**			
Among patients with no doses at randomization: New 1st dose:- patients 12+/100 patients	8.8 (8.5-9.2)<br>N patients= 101,446	8.8 (8.2-9.4)<br>N patients=30,564	1.02 (0.95-1.10), p=0.544
Among patients with 1 dose at randomization: New 2nd dose: patients 12+/100 patients	3.1 (2.9-3.3)<br>N patients=473,581	3.3 (2.9-3.7)<br>N patients=133,851	1.06 (0.93-1.20), p=0.380
Among patients with 2 doses at randomization: New 3rd dose: patients 12+/100 patients	54.8 (53.6-55.9)<br>N patients=464,682	54.7 (52.3-57.3)<br>N patients=131,356	1.00 (0.95-1.05), p=0.960
Among Pediatric patients: Number of new doses: Patients 5-11/100 patients	71.9 (68.5-75.4)<br>N patients=38,288	73.1 (65.9-81.1)<br>N patients=11,828	1.01 (1.00-1.03), p=0.150

*Poisson count model that account for clustering at the Physician Practice Level over 4 months (November 15th – March 31st). Relative rate of physicians that engaged versus control.

Adjusted for physician sex, physician years since graduation, roster size, patient sex, patient age, Patient dependency (age and labor force), Patient Ethnic concentration quintile (Racialized and newcomer populations), Patient comorbidity (Adjusted Clinical Group score)

© 2025 Shuldiner J et al. *JAMA Network Open*.
