# Supplementary material for: Practice Facilitation to Support Primary Care Physicians With COVID-19 Vaccine Uptake: A Randomized Clinical Trial
Source: JAMA Netw Open. 2025 May 14;8(5):e259967. doi: 10.1001/jamanetworkopen.2025.9967 (PMC12079287; doi:10.1001/jamanetworkopen.2025.9967)
Supplement: Supplement 3. — Data Sharing Statement [file jamanetwopen-e259967-s003.pdf]

Data Sharing Statement

Shuldiner. Practice Facilitation to Support Primary Care Physicians With COVID-19 Vaccine Uptake. *JAMA Netw Open*. Published May 14, 2025. doi:10.1001/jamanetworkopen.2025.9967

Data
Additional Information: NCT05099497
Data available: No
